# Influenza at Navy Base Hospital in France

**Published:** 1918-11

**Authors:** 


***Influenza at Navy Base Hospital in France.*** By A. W. HEWLETT, M. D. (San Francisco), and W. M. ALBERTY, M. D. (Rosedale, Kan.). *The Journal of the American Medical Association*, September 28, 1918.

The authors discuss the infectious cause of the present influenza epidemic. The etiologic agent of the disease has not been determined as yet with certainty. On account of this, the diagnosis of influenza must, for the present, be based mainly on clinical and epidemiologic characteristics.

*General Features of the Local Outbreak.* — In the lapse of a fortnight, 41 per cent. of the total personnel of the author's hospital contracted influenza. Second attacks of the disease in the same individual seemed to occur in six or seven instances, usually several weeks after the first attacks.

*Clinical Characteristics.* — The onset was sudden in some cases, and gradual in others. Headache, backache and chilliness were the initial symptoms. Dry, hacking cough occurred frequently. Mental dulness and apathy were common. The fever was usually very high, and lasted from one to six days, in uncomplicated cases. When the fever lasted more than six days, bronchitis or bronchopneumonia was present.

The pulse rate was accelerated, but not in proportion to the rise of temperature. When serious pulmonary complications were present, the pulse became rapid, thus furnishing better indications of the severity of the infection than did the temperature. Marked prostration was the rule during convalescence.

The most common complications observed were fibrinous and serofibrinous pleurisy, empyema, persistent bronchitis and bronchopneumonia. Prostration and persistent cough frequently gave rise to some suspicion of pulmonary tuberculosis; less common have been cardiac disturbances, neurasthenia and even marked and persistent general asthenia.

The treatment given was expectant and symptomatic. If the pains were troublesome acetylsalicylic acid was given in doses of

15 grains every three hours, until 6 doses had been taken. If the patient could not sleep, 5 grains of barbital were given at bed-time. Oily sprays lessened the irritation in the upper air passages. On account of the possibility of cardiac and neurasthenic sequelae, convalescence should be prolonged until the patient can carry on his usual duties without exertion.

